# Peril and pleasure: examining the impact of risk perception on behavioral intentions of mountain outdoor sports tourists

**DOI:** 10.3389/fpsyg.2026.1824943

**Published:** 2026-06-03

**Authors:** Teng Ma, Hengyi Song, Dongqi Wang, Zhiyong Zhang

**Affiliations:** 1 Faculty of Education, Qufu Normal University, Qufu, Shandong, China; 2 Graduate School of Education, Shandong Sport University, Jinan, Shandong, China; 3 School of Sports Leisure, Shandong Sport University, Jinan, Shandong, China; 4 Sports Social Science Research Institute, Shandong Sport University, Jinan, Shandong, China

**Keywords:** behavioral intention, experience quality, fuzzy set qualitative comparative analysis, mountain outdoor sports, risk perception, structural equation modeling

## Abstract

Despite growing attention to tourist risk perception in outdoor sports, little is known about how risk perception interacts with experience quality and gender differences to shape behavioral intentions from a risk marginality perspective. Addressing this gap, this study aims to examine the mechanisms linking risk perception, experience quality, and behavioral intentions, and to identify configurational pathways leading to high behavioral intentions. Drawing on the Cognitive-Affective-Behavioral Theory, this study employs a mixed-method approach combining Structural Equation Modeling (SEM) and fuzzy-set Qualitative Comparative Analysis (fsQCA). A survey of 360 mountain outdoor sports tourists with a risk preference was conducted. Results show that risk perception significantly and positively influences behavioral intentions, and experience quality partially mediates the chain “risk perception → experience quality → behavioral intentions.” Gender differences moderate these relationships: for male participants, risk perception positively affects both experience quality and behavioral intentions; for female participants, no statistically significant relationships were found. The configurational analysis identifies two distinct pathways leading to high behavioral intentions: the “Individual-Natural Risk Interwoven Type” and the “Equipment-Experience Buffering-Driven Type.” This study makes two primary contributions. Theoretically, it extends the Cognitive-Affective-Behavioral Theory by introducing the risk marginality perspective and revealing gender-differentiated mechanisms and configurational causality. Methodologically, it demonstrates the value of integrating SEM (for linear mediation and moderation) with fsQCA (for configurational pathways) in tourism safety research. For safety governance in mountain outdoor sports tourism, managers should adopt a risk marginality perspective by: (a) implementing comprehensive risk management (e.g., dynamic risk signage, graded trail systems), (b) designing experience-enhancing programs that transform risk perception into positive experiences (e.g., guided risk-exposure activities), and (c) tailoring safety measures to gender differences—especially providing more risk communication and support for female tourists.

## 1 Introduction

Mountain outdoor sports represent one of the most popular forms of outdoor activities worldwide. In 2005, the General Administration of Sport of China officially recognized and supported mountain outdoor sports as a distinct category of physical activity. In recent years, China's outdoor tourism industry has grown rapidly, with mountain outdoor sports—characterized by high engagement, immersive experiences, and thrilling physical challenges—becoming particularly attractive to outdoor enthusiasts. However, due to their inherently high-risk nature and multiple uncertain factors, accidents in mountain outdoor sports occur frequently (Yong et al., 2025). According to the *2024 China Outdoor Adventure Accident Report*, 335 outdoor adventure accidents were recorded in 2024, of which 274 occurred in mountainous areas, indicating that mountain activities account for the majority of incidents (China Adventure Association, 2025). In response, the National Development and Reform Commission and the General Administration of Sport of China jointly issued the *Guidelines on the Development of High-Quality Outdoor Sports Destinations*, emphasizing the need to comprehensively enhance safety governance, establish a sound risk prevention system, improve emergency rescue mechanisms, and raise safety awareness among participants. Thus, understanding the relationship between risk factors in mountain outdoor sports and tourist behavior is critical for addressing safety challenges at mountain tourism destinations.

In tourism research, risk perception has been widely used as an effective subjective tool for analyzing tourists' behavioral intentions (Wolff et al., 2019). However, a fundamental theoretical contradiction remains unresolved: risk perception is traditionally viewed as a negative factor that disrupts the tourist experience, yet in adventure sports contexts, it simultaneously acts as a key attraction for many participants (Barbieri and Sotomayor, 2013). Prior studies have largely treated risk perception as either a deterrent or a motivator, without reconciling this dual role. Moreover, while some scholars have focused on risk assessment and early warning systems (Zhaofang et al., 2018), few have examined the mechanisms through which tourists' subjective risk perceptions influence behavioral intentions using multi-method approaches that capture both linear and configurational effects. Most traditional quantitative studies rely on linear models (e.g., correlation or regression), which cannot reveal how different combinations of risk perception variables jointly shape behavioral intentions.

To address these gaps, this study adopts a “risk marginality” perspective—an approach that acknowledges risk as both a threat and an attraction—and integrates the Cognitive-Affective-Behavioral (CAB) theory with a mixed-method design combining Structural Equation Modeling (SEM) and fuzzy-set Qualitative Comparative Analysis (fsQCA). This study makes three key contributions. First, it extends CAB theory to the high-risk tourism context by showing how risk perception (cognitive) influences experience quality (affective) and behavioral intentions (behavioral), thereby reconciling the dual role of risk. Second, it moves beyond linear analysis by using fsQCA to identify configurational pathways (e.g., “Individual-Natural Risk Interwoven Type” and “Equipment-Experience Buffering-Driven Type”), offering a more nuanced understanding of complex interactions. Third, methodologically, it demonstrates the value of integrating SEM (for mediation and moderation) with fsQCA (for configurational causality) in adventure tourism safety research. By examining the influence of risk perception on behavioral intentions among mountain outdoor sports tourists, with experience quality as a mediator and gender as a moderator, this study aims to inform context-specific safety governance strategies for mountain outdoor tourism destinations.

## 2 Literature review

### 2.1 Theoretical framework

Existing research on adventure tourism behavior often relies on a single theoretical lens. On one hand, edgework theory suggests that individuals voluntarily engage in high-risk activities because they perceive and accept the risks associated with marginal states, seeking emotional stimulation and affective gratification as key drivers for continued participation (Laurendeau, 2006; Lyng, 1990). On the other hand, the Cognitive-Affective-Behavioral (CAB) theory explains how individuals make behavioral decisions through the interplay of cognition and emotion (Muehling et al., 2004). However, these two perspectives remain largely disconnected: edgework theory explains why individuals actively seek risk but does not clarify the specific psychological pathway from risk perception to behavioral intention; CAB theory provides a linear cognitive → affective → behavioral path but fails to explain how risk transforms from a “threat” into an “attraction” in adventure contexts.

The theoretical contribution of this study lies in integrating edgework and CAB theories. Specifically, edgework theory provides the motivational basis for why risk perception may have a positive effect in adventure tourism—individuals actively pursue the excitement generated by edge experiences. CAB theory then reveals the underlying mechanism: risk perception (cognitive) influences behavioral intention (behavioral) through perceived experience quality (affective). This integration not only bridges the blind spots of each theory but also addresses the theoretical contradiction that “risk is both a deterrent and an attraction” in adventure tourism. Additionally, complexity theory provides a framework for understanding the nonlinear interactions and dynamic evolution of multidimensional elements within complex systems. It helps explain how different risk variables jointly shape behavioral intentions through configurational pathways and reveals feedback mechanisms and counterfactual cases (Meiyu et al., 2024; Pappas et al., 2016). This study adopts complexity theory as a methodological supplement to justify the subsequent use of fsQCA for configurational analysis.

### 2.2 Risk perception among mountain outdoor sport tourists

Risk perception refers to an individual's subjective feelings and cognitive assessments of objectively existing potential dangers, often used to measure psychological tension or anxiety. In tourism research, risk perception is regarded as a personal evaluation of the uncertainties inherent in the travel experience and its potential consequences (Wolff et al., 2019). As a multi-level construct, tourism risk perception encompasses multiple dimensions. To date, there is no universally accepted classification; scholars typically adopt different typologies based on their research objectives and target populations (Yong et al., 2025; Lepp and Gibson, 2003). Classical models commonly include eight dimensions: physical health, facilities and equipment, financial security, psychological needs, time costs, political conditions, social interactions, and expectation fulfillment.

For mountain outdoor sports, existing studies have identified major risk sources: the natural environment, activity characteristics, management and organizational factors, and tourists' own physical conditions (Zhaofang et al., 2018). However, most studies treat risk perception as a monolithic variable, neglecting the differential effects of distinct risk dimensions on behavioral intentions. For example, natural risk (e.g., adverse weather) may inhibit behavioral intention, while individual risk (e.g., physical challenge) may enhance it. This within-dimension contradiction has not been critically examined in the literature. Therefore, this study focuses on the inherent risks of mountain outdoor sports and, drawing on established dimensions of tourism risk perception, categorizes tourists' perceived risks into four dimensions: individual risk (threats to physical or psychological wellbeing), natural risk (environmental hazards inherent in mountainous terrain), social risk (risks from interactions with team members, organizers, or local residents), and equipment risk (concerns about quality, suitability, and safety of equipment). This four-dimensional categorization facilitates subsequent fsQCA analysis of configurational effects.

### 2.3 The influence of risk perception on behavioral intention

Behavioral intention refers to an individual's predisposition toward engaging in a specific behavior and is a key predictor of future tourist behavior, typically including recommendation and revisit intentions (Tavitiyaman and Qu, 2013). In conventional tourism settings, risk perception is generally viewed as a suppressive factor. However, in high-adventure sports tourism contexts, it may instead stimulate challenge motivation and emotional engagement (Jincheng and Hai, 2020). This contradiction raises a central research question: Why does the same risk perception produce opposite effects across different contexts?

Integrating edgework and CAB theories provides an explanation. Edgework theory argues that individuals who seek edge experiences actively pursue the excitement that risk brings, and this excitement constitutes a positive affective response (Lyng, 1990). Thus, in adventure sports tourism, risk perception is no longer merely a threat appraisal but also a positive challenge signal. Empirical studies have shown that in adventure sports tourism settings, risk perception has a significant positive effect on tourists' emotional arousal (Yangle and Wenxin, 2023). Compared with general tourism contexts, mountain outdoor sports represent a form of adventure tourism where risk serves as a core attraction. Risk-oriented individuals tend to perceive and accept the risks associated with marginal experiences. Such “edge” experiences not only strengthen their identification with the activity but also enhance their intention to revisit or recommend it. Based on the above theoretical integration and empirical support, the following hypothesis is proposed:

**H1:** Risk perception has a significant positive effect on behavioral intention.

### 2.4 The mediating role of perceived experience quality

Perceived experience quality generally refers to an individual's overall evaluation of an activity, service, or product, involving perceived service level, satisfaction, and the gap between expectations and actual experience (Lemke et al., 2011). From a tourism experience perspective, perceived experience quality is considered an antecedent of behavioral intention. Incorporating this construct into tourist behavioral models significantly improves explanatory power (Moon and Han, 2019). High-quality experience not only enhances cognitive evaluation of the activity but also stimulates behavioral intention through positive affective responses (Yang and Xuegang, 2023).

In this study, perceived experience quality is positioned as the affective bridge between risk perception and behavioral intention. According to the integrated theoretical framework: edgework theory explains why risk perception can generate positive affect (excitement, sense of accomplishment); CAB theory provides the cognitive → affective → behavioral pathway. Previous research has shown that perceived experience quality is partly influenced by tourists' cognitive appraisal of risk and mediates the relationship between risk perception and behavioral intention (Fave et al., 2003; Yangle and Wenxin, 2023; Yüksel and Yüksel, 2007). When tourists perceive an activity as both safe and challenging, and derive enjoyment and a sense of accomplishment, this heightened risk perception contributes to a richer, higher-quality sport experience. Therefore, the following hypotheses are proposed:

**H2:** Risk perception has a significant positive effect on perceived experience quality.
**H3:** Perceived experience quality has a significant positive effect on behavioral intention.
**H4:** Perceived experience quality mediates the relationship between risk perception and behavioral intention.

### 2.5 Group differences between genders

Due to physiological and psychological differences, women are more likely to encounter various risks. Numerous studies have demonstrated gender differences in both risk perception and risk-taking behavior. Women tend to exhibit higher sensitivity and greater susceptibility to external influences (Berdychevsky and Gibson, 2015; Yang et al., 2017). Men generally place greater emphasis on the challenge and sense of achievement associated with sports experiences, exhibiting higher risk-taking tendencies and stronger risk tolerance, which often results in greater emotional satisfaction in high-risk environments (Kim and Seo, 2019). In contrast, women tend to prioritize emotional fulfillment and safety during physical activities. When exposed to the same risks, they often show heightened risk sensitivity, which may lead to increased anxiety and insecurity, and a greater likelihood of adopting protective strategies or avoiding high-risk situations (Brown et al., 2020; Park and Reisinger, 2010).

However, most existing studies treat gender as a control variable rather than a moderator, failing to systematically test its moderating role in the chain of “risk perception → perceived experience quality → behavioral intention.” This study positions gender as a moderator, examining its differential effects on all three paths and addressing the question: Why are men more likely than women to translate risk perception into positive experiences and behavioral intentions? Based on the above analysis, the following hypotheses are proposed:

**H5:** There are significant gender-based differences in the impact of risk perception on behavioral intention.
**H6:** There are significant gender-based differences in the impact of risk perception on perceived experience quality.
**H7:** There are significant gender-based differences in the impact of perceived experience quality on behavioral intention.

**Figure 1:**
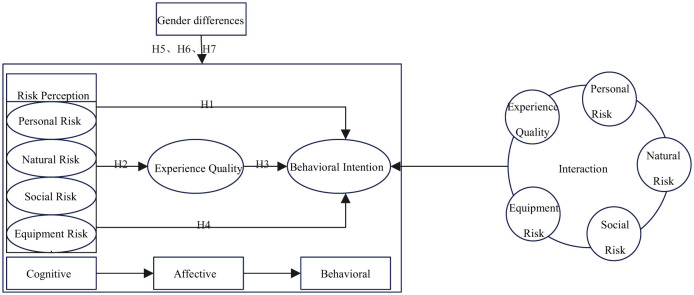
Theoretical model.

Based on the aforementioned theoretical derivations and research hypotheses, this study constructs a theoretical model illustrating the influence of risk perception on behavioral intention among mountain outdoor sport tourists (see Figure 1).

## 3 Research design

### 3.1 Questionnaire design

The questionnaire consisted of three sections. The first section served as a screening filter, using the question “Do you enjoy adventure?” to ensure that respondents were risk-oriented mountain outdoor sports tourists. The second section collected demographic information, including gender, age, marital status, educational background, and monthly income. The third section comprised measurement scales for risk perception, perceived experience quality, and behavioral intention, all rated on a 5-point Likert scale. The risk perception scale was based on the risk assessment framework for mountain outdoor sports developed by Peng Zhaofang et al., and informed by the studies of Chen Yangle et al. and She et al. It encompasses four dimensions—individual, natural, social, and equipment risks—and consists of 20 items. The perceived experience quality scale was adapted from Zhao Yang et al.'s work and includes four items. The behavioral intention scale was derived from Tu Hongwei et al.'s research, covering recommendation and revisit intentions with a total of four items (Hongwei et al., 2017; She et al., 2019; Yang and Xuegang, 2023; Yangle and Wenxin, 2023; Zhaofang et al., 2018).

### 3.2 Data collection

Fieldwork was conducted in Siguniang Mountain, Sichuan Province, from January 11 to 17, 2025, targeting tourists who were actively participating in mountain outdoor sports. In collaboration with staff from the Sichuan Mountaineering Association and professionals in mountain outdoor sports, questionnaires were administered across various mountain sport venues, tourist gathering points, and time slots. Trained personnel guided respondents to ensure coverage of diverse sport modalities and demographic profiles, thereby enhancing sample heterogeneity and representativeness. Initially, a pilot survey was conducted on a small scale to assess the questionnaire's reliability; the instrument was refined accordingly before the main field survey. Subsequently, 408 questionnaires were distributed during the formal survey. After excluding invalid responses, 360 valid questionnaires were retained, yielding an effective response rate of 88.2%. The sample size exceeded 10 times the number of scale items, satisfying the requirements for subsequent data analyses. Demographic analysis indicated that 58.6% of respondents were male, reflecting a predominance of male participants in mountain outdoor sports. The majority (89.7%) were aged between 18 and 41 years, indicating a primarily young-to-middle-aged cohort. Individuals with a tertiary education or higher (including vocational college) accounted for 82.7%, suggesting a generally high educational level. Unmarried respondents comprised 70.8% of the sample, and 57.5% reported a monthly income above Chinese Yuan (CNY) 5,000, indicative of a relatively high-income group (see Table 1). The sample structure aligns well with the characteristics of mountain outdoor sport tourists, demonstrating strong representativeness.

**Table 1:** Basic characteristics of survey sample.

Category	Option	Count	Percentage
Gender	Male	211	58.6
	Female	149	41.4
Age	< 18	9	2.5
	18–25	170	47.2
	26–30	87	24.2
	31–41	66	18.3
	41–50	20	5.6
	51–60	7	1.9
	>60	1	0.3
Education	Junior High School or Below	14	3.9
	Senior High School/Technical Secondary School	48	13.3
	Junior College/Associate Degree	106	29.4
	Bachelor's Degree	151	41.9
	Master's Degree or Above	41	11.4
Marital Status	Single	255	70.8
	Married	105	29.2
Monthly Income	< 2,000 CNY	49	13.6
	2,000–5,000 CNY	104	28.9
	5,001–10,000 CNY	147	40.8
	>10,000 CNY	60	16.7

This study was conducted in accordance with the Declaration of Helsinki. Approval was obtained from the Sports Science Ethics Committee of Shandong Sport University (Approval No.: 2024067). Before data collection, all participants were fully informed of the study's purpose, the voluntary nature of their participation, and their right to withdraw at any time without penalty. Informed consent was obtained from each respondent prior to the questionnaire administration (verbal consent/online click consent). Anonymity and confidentiality of responses were strictly guaranteed, and no personally identifiable information was collected. All data were used solely for academic purposes. The fieldwork was conducted in a single mountainous region and during a specific time window, which may limit geographic and seasonal generalizability.

### 3.3 Research methods

This study employs Structural Equation Modeling (SEM) and fuzzy-set Qualitative Comparative Analysis (fsQCA) sequentially to investigate the determinants and configurational pathways through which risk perception influences behavioral intention. SEM focuses on estimating the net effects of independent variables on dependent variables, but it is limited to linear relationships and cannot capture how different risk dimensions combine to produce high behavioral intentions. In contrast, fsQCA uses set theory and Boolean algebra to analyze combinatorial causal pathways of antecedent conditions. It is particularly suited for this study because: (a) risk perception comprises four distinct dimensions (individual, natural, social, equipment) that may interact nonlinearly; (b) different combinations of these dimensions may lead to the same outcome (equifinality); and (c) fsQCA allows for asymmetric relationships—that is, the absence of high risk perception does not necessarily imply low behavioral intention. Therefore, integrating SEM (for linear mediation and moderation effects) with fsQCA (for configurational effects) provides a more complete understanding.

SPSS 27.0 (IBM Corp., Armonk, NY, USA) and AMOS 24.0 (IBM Corp., Armonk, NY, USA) were used to assess normality and to conduct reliability and validity analyses. Confirmatory factor analysis (CFA) was performed to confirm the measurement scales' consistency and construct validity. Next, the SEM was constructed using the maximum likelihood estimation method. Direct and mediating effects were tested via bias-corrected percentile bootstrap procedures with 5,000 resamples and a 95% confidence interval. In addition, multi-group comparisons across male and female tourist samples were performed using AMOS 24.0. Finally, fsQCA 3.0 (University of California, Irvine, CA, USA) was employed following the sequence of calibration, necessity analysis, and configurational analysis to examine the combined effects of antecedent conditions and to explore the mechanisms underlying behavioral intention across different causal pathways.

## 4 Research results

### 4.1 Common method bias and data normality tests

The CFA-based Harman single-factor test yielded χ^2^ = 3,914.54, df = 350, χ^2^/df = 11.18, *CFI* = 0.557, *IFI* = 0.558, *TLI* = 0.521, *RMSEA* = 0.168, *SRMR* = 0.143. All fit indices fell below the acceptable thresholds, indicating that a single-factor model did not fit the data. As an additional robustness check, the full collinearity Variance Inflation Factor (VIF) method was employed. The results showed that all VIF values ranged from 1.026 to 3.053, all below the recommended threshold of 3.3. This further confirms that common method bias does not pose a serious threat to the validity of the findings. The normality assessment showed that the absolute values of kurtosis coefficients were well below 10 and those of skewness coefficients were within ±3, supporting an approximately normal distribution of the data.

### 4.2 Reliability and validity tests

During the CFA phase, a second-order model was specified to simplify the measurement structure, in which individual, natural, social, and equipment risks served as first-order factors subsumed under the second-order factor of risk perception. To ensure that risk perception appropriately functions as a second-order construct, first-order CFA must be conducted and inter-dimension correlations examined before proceeding to the second-order analysis. Only if all first-order model fit indices meet established criteria will the analysis advance to the second-order CFA stage. The first-order CFA results indicated good model fit for the measurement models of individual risk, natural risk, social risk, equipment risk, perceived experience quality, and behavioral intention. The second-order CFA further demonstrated satisfactory fit for the higher-order constructs of risk perception, perceived experience quality, and behavioral intention (see Table 2). Additionally, following Doll et al.'s target-coefficient (T) approach, the chi-square of the fully correlated first-order model was compared with that of the second-order model; the closer *T* is to 1, the better the second-order model replicates the first-order structure (Doll et al., 1994). The resulting *T*-value was 0.949 (974.34 / 1,026.88), indicating that the second-order model accounts for 94.9% of the first-order structure and thus satisfies the theoretical requirements for treating risk perception as a second-order construct.

**Table 2:** Model fit index results.

Measurement model/fit indices	χ^2^	df	χ^2^/df	*CFI*	*IFI*	*TLI*	*RMSEA*	*SRMR*
			≤ 3	≥0.900	≥0.900	≥0.900	≤ 0.080	≤ 0.080
First-order model	974.34	335	2.908	0.921	0.921	0.910	0.073	0.044
Second-order model	1,026.88	343	2.994	0.915	0.915	0.906	0.075	0.052

Cronbach's *a* coefficients were calculated using SPSS 27.0 to assess the internal consistency of the survey data. The results indicated that Cronbach's *a* for risk perception, perceived experience quality, behavioral intention, and its sub-dimensions—individual risk, natural risk, social risk, and equipment risk—all exceeded 0.90. Composite reliability (CR) values also surpassed 0.90, meeting the established reliability criteria. Convergent validity was evaluated by computing the Average Variance Extracted (AVE). AVE values for risk perception, perceived experience quality, and behavioral intention all exceeded 0.60, and all factor loadings were above 0.70, indicating satisfactory convergent validity (see Table 3). The square roots of the AVE for each latent construct ranged from 0.81 to 0.85 and exceeded the Pearson correlations between constructs, demonstrating adequate discriminant validity (see Table 4).

**Table 3:** Internal consistency, convergent validity, and combinatorial reliability.

Construct	Item code	Statement	Cronbach's *a*	Factor loading	CR	AVE
Risk perception	IRP	Personal risk	0.958	0.826	0.912	0.721
	NRP	Natural risk		0.913		
	SRP	Social risk		0.874		
	ERP	Equipment risk		0.777		
Personal risk	IRP1	I may find it difficult to cope with the intensity of the activity due to poor physical fitness.	0.912	0.787	0.913	0.678
	IRP2	My health problems may affect my sports experience.		0.803		
	IRP3	Psychological stress or anxiety may impair my judgment.		0.827		
	IRP4	I may lack the ability to respond to emergencies (e.g., getting lost or injured).		0.831		
	IRP5	I may be unable to complete the activity due to physical or psychological limits.		0.868		
Natural risk	NRP1	Extreme weather (e.g., heavy rain, lightning) may occur.	0.922	0.856	0.923	0.706
	NRP2	Geological disasters (e.g., landslides, mudslides) may happen.		0.88		
	NRP3	High-altitude environments (e.g., hypoxia, strong UV rays) may pose risks.		0.838		
	NRP4	I may encounter attacks from wild animals or plants.		0.81		
	NRP5	Safety facilities in the environment may be inadequate.		0.816		
Social risk	SRP1	I may have misunderstandings or disagreements with team members.	0.91	0.838	0.911	0.672
	SRP2	Team members may lack cooperation or violate rules, affecting safety.		0.862		
	SRP3	The decision-making and command skills of the leader/organizer may be insufficient.		0.84		
	SRP4	Unfriendly attitudes from local residents or service staff may affect my experience.		0.808		
	SRP5	I may feel isolated or neglected due to a lack of team integration.		0.743		
Equipment risk	ERP1	The equipment may have quality issues.	0.904	0.819	0.905	0.657
	ERP2	The equipment may not meet the requirements of the activity.		0.842		
	ERP3	I may not be skilled enough in using the equipment correctly.		0.793		
	ERP4	Damaged equipment may not be replaced in time.		0.829		
	ERP5	Inadequate equipment maintenance may cause safety hazards.		0.767		
Experience quality	EQ1	I found the activity interesting and gained profound experiences.	0.906	0.812	0.907	0.708
	EQ2	The facilities and services during the activity made me feel comfortable and exceeded my expectations.		0.843		
	EQ3	The activity helped me relax physically and mentally, making me feel happy and fulfilled.		0.876		
	EQ4	The overall design of the activity was scientific and reasonable, fully meeting my needs.		0.834		
Behavioral intention	BI1	I am willing to participate in mountain outdoor sports again.	0.907	0.833	0.908	0.711
	BI2	I will recommend others to participate in mountain outdoor sports.		0.852		
	BI3	I will encourage others to participate in mountain outdoor sports.		0.847		
	BI4	I plan to participate in mountain outdoor sports again.		0.842		

**Table 4:** Discriminant validity.

Variable	Discriminant validity test based on factor correlations and square root of AVE			
	1	2	3	4
EQ	0.842			
BI	0.391	**0.843**		
RP	0.149	0.301	**0.849**	
IRP	**0.824**			
NRP	0.802	**0.84**		
SRP	0.674	0.783	**0.82**	
ERP	0.61	0.657	0.783	**0.81**

The bold values on the diagonal represent the square root of the Average Variance Extracted (AVE). EQ, Experience Quality; BI, Behavioral Intention; RP, Risk Perception; IRP, Individual Risk; NRP, Natural Risk; SRP, Social Risk; ERP, Equipment Risk. Same abbreviations apply hereafter.

### 4.3 Hypothesis testing

#### 4.3.1 Tests of direct and mediating effects

The structural equation model yielded χ^2^ = 1,026.875, df = 343, χ^2^/df = 2.994, *CFI* = 0.915, *IFI* = 0.915, *TLI* = 0.906, *RMSEA* = 0.075, *SRMR* = 0.052, indicating an acceptable model fit. Tests of direct and mediating effects showed that the direct effect of risk perception on behavioral intention was significant [β = 0.248, *p* < 0.01, 95% *CI* (0.127, 0.368)], supporting H1. The direct effect of risk perception on perceived experience quality was significant [β = 0.149, *p* < 0.05, 95% *CI* (0.010, 0.287)], supporting H2. The direct effect of perceived experience quality on behavioral intention was significant [β = 0.354, *p* < 0.01, 95% *CI* (0.225, 0.488)], supporting H3. Moreover, using both bias-corrected and percentile bootstrap methods, the indirect path “risk perception → perceived experience quality → behavioral intention” had a 95% confidence interval that did not include zero and was statistically significant at the 5% level [β = 0.053, *p* < 0.05, 95% *CI* (0.005, 0.126)], indicating that perceived experience quality partially mediates the effect of risk perception on behavioral intention. Specifically, the total effect of risk perception on behavioral intention was 0.301, with an indirect effect of 0.053 via perceived experience quality, accounting for 17.6% of the total effect, thereby confirming H4 (see Table 5).

**Table 5:** Direct effect and mediation effect test results.

Path relationships	Standardized path coefficient	S.E.	Bootstrap test					
			Bias-corrected 95% CI			Percentile 95% CI		
			Lower	Upper	*P*	Lower	Upper	*P*
H1:RP → BI	0.248	0.061	0.127	0.368	0.000	0.122	0.362	0.000
H2:RP → EQ	0.149	0.071	0.010	0.287	0.031	0.007	0.281	0.036
H3:EQ → BI	0.354	0.067	0.225	0.488	0.000	0.217	0.478	0.000
H4:RP → EQ → BI	0.053	0.030	0.005	0.126	0.023	0.002	0.120	0.036
Total effect	0.301	0.065	0.173	0.428	0.000	0.169	0.424	0.000

#### 4.3.2 Multi-group comparison analysis

Multi-group comparison analyses were conducted using AMOS 24.0 for male and female cohorts. First, all dimensions of risk perception (individual, natural, social, and equipment risks) were treated as observed indicators for their respective latent constructs. Then, the structural equation model “risk perception → perceived experience quality → behavioral intention” was estimated separately for each gender group. The comparison across nested models revealed that, except for the measurement-weights model, all alternative models (including the structural-weights, structural-covariances, structural-residuals, and measurement-residuals models) exhibited *p*-values below 0.05, indicating significant differences in path coefficients within the measurement model. As shown in Table 6, for male participants, risk perception had significant positive effects on both perceived experience quality (β = 0.265, *p* < 0.001) and behavioral intention (β = 0.382, *p* < 0.001), and perceived experience quality significantly predicted behavioral intention (β = 0.488, *p* < 0.001). For female participants, none of these paths reached statistical significance (all *p* > 0.05). Thus, H5, H6, and H7 were supported (see Table 6).

**Table 6:** Group difference analysis results.

Path relationships	Male			Female		
	Standardized path coefficient	S.E.	C.R.	Standardized path coefficient	S.E.	C.R.
H5:RP → BI	0.382^***^	0.076	5.052	0.083	0.107	0.773
H6:RP → EQ	0.265^***^	0.079	3.35	−0.012	0.099	−0.124
H7:EQ → BI	0.488^***^	0.074	6.63	0.067	0.098	0.684

^***^ indicates statistical significance at the *p* < 0.001 level.

The stark contrast between male and female results warrants careful interpretation. Several factors may contribute to this pattern. First, the sample included 211 males and 149 females; although adequate for multi-group analysis, the smaller female subsample may have reduced statistical power to detect significant effects. Second, measurement invariance across genders was not formally tested; if the factor structure differs between groups, direct comparisons of path coefficients may be biased. Third, social desirability or culturally shaped risk expressions might influence self-reported risk perception and behavioral intentions. These findings suggest that gender moderates the risk–experience–intention relationship, but the extreme nature of the difference (all significant for males, none for females) should be interpreted with caution. We revisit these issues in the Section. 5.

### 4.4 Fuzzy-set qualitative comparative analysis (fsQCA)

#### 4.4.1 Calibration of variables

Based on the literature review and empirical findings, five variables—individual risk, natural risk, social risk, equipment risk, and perceived experience quality—were selected as condition variables, with behavioral intention serving as the outcome variable. Prior to configurational analysis, all latent variables were transformed into their observed counterparts. Next, because fsQCA requires variable values to lie between 0 and 1, traditional calibration typically employs three anchor points (1, 3, and 5) to rescale raw data. However, this approach overlooks the empirical distribution of the data. Therefore, this study calibrated variables by setting the 95th percentile as the threshold for full membership, the 50th percentile as the crossover point, and the 5th percentile as the threshold for full non-membership. These anchors were applied in fsQCA 3.0 to convert each variable into fuzzy-set scores between 0 and 1 (Fiss, 2011). Additionally, to include all cases in the analysis, any score equal to 0.5 was adjusted to 0.501 prior to subsequent fsQCA procedures (Crilly et al., 2012).

#### 4.4.2 Necessity analysis

Before proceeding to configurational analysis, the necessity of each condition variable (including its negation) for the outcome variable was assessed to determine whether any could be considered necessary for behavioral intention. The necessity analysis revealed that the consistency values for all condition variables were below 0.90 (see Table 7), indicating that none constitute necessary conditions for either high or low behavioral intention. Therefore, a configurational analysis of condition combinations is warranted (Fiss, 2011).

**Table 7:** Analysis results of necessary conditions.

Conditional variable	BI		~**BI**	
	Consistency	Coverage	Consistency	Coverage
IRP	0.671781	0.770167	0.646235	0.548188
~IRP	0.605906	0.698320	0.729060	0.621720
NRP	0.704636	0.786562	0.641800	0.530090
~NRP	0.579035	0.686001	0.741583	0.650072
SRP	0.667092	0.758026	0.663414	0.557782
~SRP	0.610831	0.710371	0.712201	0.612842
ERP	0.641260	0.752449	0.656294	0.569801
~ERP	0.633372	0.713511	0.714872	0.595870
EQ	0.738560	0.779653	0.667974	0.521744
~EQ	0.546952	0.690054	0.717897	0.670159

“~” denotes the set relation of “non-” or negation.

#### 4.4.3 Configurational analysis

Following prior studies (Fiss, 2011), the case frequency threshold was set to 2, the raw consistency threshold to 0.8, and the Proportional Reduction in Inconsistency (PRI) consistency threshold for high behavioral intention to 0.7. The configurational analysis identified three pathways associated with high behavioral intention (A1, A2a, A2b), with consistencies of 0.886, 0.895, and 0.904, respectively. The overall solution consistency was 0.872, and solution coverage was 0.551, indicating that the three pathways jointly explain 55.1% of high behavioral intention cases (see Table 8). Based on the configurations of antecedent conditions, the three pathways were classified into two types: Individual–Natural Risk Intertwining Type (A1) and Equipment–Experience Buffering Type (A2a, A2b).

**Table 8:** Results of configuration analysis.

Predictor variable	High behavioral intention		
	A1	A2a	A2b
IRP	•		•
NRP	•	•	
SRP			•
ERP		⊗	⊗
EQ	•	•	•
Consistency	0.886253	0.894502	0.904286
Raw coverage	0.501815	0.374096	0.319632
Unique coverage	0.164422	0.036703	0.012093
Solution consistency		0.872313	
Solution coverage		0.55061	

Path A1 (Individual–Natural Risk Intertwining). This pathway is defined by the presence of individual risk, natural risk, and perceived experience quality. It accounts for 50.18% of the sample (raw coverage = 0.502). The consistency is 0.886. This configuration suggests that when tourists perceive both personal vulnerabilities and environmental hazards, high experience quality is critical for achieving high behavioral intention.

Paths A2a and A2b (Equipment–Experience Buffering). These pathways share the combination of low equipment risk and high perceived experience quality, with different additional conditions. Path A2a (natural risk ^*^ ~equipment risk ^*^ experience quality) accounts for 37.41% of the sample (raw coverage = 0.374, consistency = 0.895). Path A2b (individual risk ^*^ social risk ^*^ ~equipment risk ^*^ experience quality) accounts for 31.96% of the sample (raw coverage = 0.320, consistency = 0.904). In both paths, low equipment risk and high experience quality jointly contribute to high behavioral intention, regardless of whether natural or social risks are present.

## 5 Discussion

### 5.1 Dimensional classification and analysis of risk perception among mountain outdoor sports tourists

The dimensions of risk perception are characterized by situational specificity and individual variability. Accordingly, this study divides mountain-based outdoor adventure tourists' risk perception into four primary dimensions—individual, natural, social, and equipment risks—and verifies their applicability. From the perspective of edgework theory, mountain outdoor adventure is essentially an activity in which individuals voluntarily pursue boundary experiences. These four dimensions correspond exactly to four core types of uncertainty that adventure tourists face: the limits of personal competence, the forces of the natural environment, tensions in social interaction, and the reliability of technical equipment.

The individual risk dimension shares similarities with physical and psychological risk (Yangle and Wenxin, 2023) but is further integrated and expanded within the mountain adventure context. High-intensity and extreme activities require tourists to endure not only physiological stress but also psychological experiences such as fear and anxiety, which precisely reflects the continuous testing and transcendence of physical and mental boundaries emphasized by edgework theory. The natural risk dimension has received extensive attention in previous research (Yangle and Wenxin, 2023). In mountain outdoor adventure, it manifests more saliently as the uncontrollability and challenge of the environment, including sudden weather changes, terrain obstacles, and wildlife. These factors embody the force of the “other” in boundary experiences; tourists gain a sense of control and peak experience by voluntarily encountering and coping with natural risks. The social risk dimension lacks a clear definition in existing studies, and scholars often interpret it according to specific situational contexts (Yanbo and Pingping, 2019). Mountain sports are frequently conducted through teamwork and group interaction, where tourists may perceive uncertainties arising from interpersonal relationships or organizational management during interactions with organizers, local residents, and team members. This essentially constitutes a social boundary experience in edgework. The equipment risk dimension differs from the facility risk found in traditional tourism (Yanbo and Pingping, 2019; Yangle and Wenxin, 2023). Mountain outdoor adventure tourists are highly dependent on personal gear, equipment provided by organizers, and rented equipment. The performance, suitability, and potential hazards in the use of such equipment directly affect the balance between safety and challenge, reflecting the high degree of reliance on technological objects and the risks that accompany them in edgework.

### 5.2 Mechanisms of the influence of risk perception on behavioral intentions among mountain outdoor sports tourists

The study found that mountain outdoor adventure tourists' risk perception had a significant positive total effect on behavioral intention, an effect realized through both the direct path and the mediating role of experience quality. This seemingly counterintuitive result can be precisely explained by edgework theory and cognitive-affective-behavioral (CAB) theory. Edgework theory posits that active risk-takers do not avoid risk; rather, through skill, focus, and situational control, they transform risk into a highly compelling boundary experience. In this process, risk perception plays the role of a cognitive activator, signaling environmental challenges to the individual, while experience quality serves as an affective catalyst, converting this cognitive excitement into immersion and a sense of accomplishment, which ultimately drives positive behavioral intention. This pathway aligns closely with the CAB framework of “cognition → affect → behavior.”

This helps explain why the present finding differs from some previous studies. For example, Yangle and Wenxin (2023) studied risk-preference individuals and found that risk perception had no significant effect on revisit intention in a surfing tourism context; Yong et al. (2025) reported a significant negative effect of risk perception on behavioral intentions among mountaineering tourists; and Jing et al. (2015) found that tourism risk perception could positively influence destination loyalty. The divergence of these findings may be related to the research context and the type of tourists involved. Such inconsistencies likely stem from differences in the depth of boundary experience and the heterogeneity of participants' risk preferences across contexts. The inherently high adventure characteristics of mountain outdoor activities and tourists' strong risk preferences make it easier for risk perception to remain within an “optimal arousal” zone, thereby generating a positive driving force—rather than inhibiting behavioral intention—under the catalysis of experience quality.

Therefore, risk perception is not a purely negative factor. When risk perception remains at an appropriate level, the complex cognitive-affective interaction between risk cognition and experience quality can effectively stimulate tourists' intentions to participate and recommend. Management and planning practice should not aim to eliminate risk; instead, drawing on the ideas of edgework theory, it should respect tourists' risk preferences while providing appropriate safety measures, skill training, and guidance. This creates a form of “bounded safety stimulus” and helps tourists obtain high-quality edgework experiences through self-control and challenge.

### 5.3 Multi-group analysis of mountain outdoor sports tourists by gender

There is a significant gender difference in the relationship among risk perception, experience quality, and behavioral intention. In the male group, risk perception exerts a significant positive effect on both experience quality and behavioral intention, whereas the corresponding paths in the female group are not significant. This stark contrast requires a deeper interpretation beyond descriptive statements.

According to edgework theory, the propensity to seek and enjoy risk is socially gendered. In many societies, males are socialized from an early age to embrace risk as a test of competence, courage, and masculinity, whereas females are often socialized to prioritize safety, care, and risk avoidance (Kim and Seo, 2019). This cultural script shapes how men and women interpret the same risk cues: men tend to view risk as a challenge to overcome, while women may perceive it as a threat to personal safety. Consequently, in mountain outdoor sports, men's risk perception is more likely to translate into positive affect and behavioral intentions, whereas women's risk perception remains uncoupled from these outcomes.

Meanwhile, the four dimensions of risk perception (individual, natural, social, equipment) may have different salience or factor structures for men and women. For example, women might be more sensitive to social risk (e.g., team conflicts, lack of support) and equipment risk (e.g., poorly maintained gear), while men focus more on individual and natural risks (Brown et al., 2020). In our study, the linear path model collapsed all four dimensions into a single second-order factor. If women's risk perception is driven primarily by social or equipment risks, which may not enhance experience quality as directly as individual or natural risks do, the overall effect could be diluted.

Furthermore, women may use response scales differently from men—for instance, showing greater reluctance to endorse extreme values or being more cautious when reporting risk-related feelings. Such measurement differences could attenuate observed path coefficients in the female subsample. We did not formally test differential item functioning across genders.

Although this study demonstrates that gender moderates the relationship among risk perception, experience quality, and behavioral intention, the absence of significant paths for females does not prove that risk perception is irrelevant for female tourists. Rather, it may operate through different risk dimensions or under specific configurational conditions. Therefore, management strategies for mountain outdoor adventure destinations should fully incorporate a gender perspective. For male tourists, emphasize challenge and achievement; for female tourists, provide stronger safety assurances and social support, and explore pathways involving the reduction of social or equipment risks.

### 5.4 Configurational analysis of factors influencing the behavioral intentions of mountain outdoor sports tourists

The fsQCA configurational analysis further reveals that the formation of high behavioral intention is not independently driven by a single factor; rather, it is the product of the synergistic interaction of multiple conditions—individual risk, natural risk, social risk, equipment risk, and experience quality. This fully reflects the characteristic of equifinality in causal complexity, whereby different combinations of antecedent conditions can achieve the same outcome through differentiated mechanisms. This not only compensates for the limitation of traditional regression analysis in handling multi-factor conjunctural effects, but also deepens the conceptual understanding of risk perception effects (Runze et al., 2024). Experience quality appears as a core condition in all configurational paths, underscoring its critical catalytic role in the risk–behavior transformation. According to the CAB model, experience quality functions as the affective hub linking risk cognition to behavioral response, enabling risk perception to be converted into positive behavioral drive. The different paths, in turn, demonstrate the multiple possibilities behind equifinality: in some paths, high risk perception co-occurs synergistically with high experience quality, reflecting a typical pattern in which edgeworkers gain immersive experience under full-spectrum challenges and generate strong behavioral intention; in other paths, certain risk dimensions are absent or not salient, yet high experience quality still combines with other conditions to produce high behavioral intention, indicating that even in contexts of lower risk perception, superior affective experience can play a substitute driving role. These findings highlight the explanatory power of edgework theory in configurational analysis. High behavioral intention can be realized under different combinations of risk conditions, not confined to the minimization or maximization of risk, but contingent on the asymmetrical matching and synergy between risk conditions and experience quality. This suggests that managers need not pursue a uniform “low-risk” model; instead, they should flexibly allocate resources according to different condition configurations—for example, emphasizing service interaction to enhance experience quality when natural risk is low, or providing emotional support and technical training when social risk is high—so as to realize multiple pathways to high participation intention. Such an equifinality-based pluralistic strategy offers insight for the governance of mountain outdoor adventure destinations from the perspective of boundary management in edgework theory.

### 5.5 Theoretical contribution

The theoretical contributions of this study are mainly manifested in the following three aspects.

First, this study extends the cognitive-affective-behavioral (CAB) theoretical framework to the high-risk context of mountain outdoor adventure and reveals the dual mechanism of risk perception. Traditional tourism research has largely treated risk as a negative factor to be eliminated. In contrast, this study finds that risk perception exerts a significant positive total effect on behavioral intention through the mediating role of experience quality. This indicates that in edgework, where individuals voluntarily pursue boundary experiences, risk not only produces inhibitory effects but also plays an important role as a cognitive activator. Drawing on edgework theory, this study further clarifies that active risk-takers do not avoid risk; rather, by building on skill and situational control, they transform moderate risk into a strongly compelling affective experience, which in turn drives intentions to participate and recommend. This finding revises the operative pathway of the CAB model in high-risk contexts, provides a theoretical basis for understanding the conditions under which risk perception acts as a barrier and the conditions under which it transforms into a driving force, and opens up a dialogue between cognitive appraisal theories and affect-driven theories for future research.

Second, moving beyond the traditional linear analytical framework, this study employs the fsQCA method to identify multiple configurational paths leading to high behavioral intention, thereby reinforcing the theoretical implications of causal complexity and equifinality. The differentiated configurations identified—such as the “individual-natural risk intertwined type” and the “equipment-experience buffering driven type”—demonstrate that high behavioral intention does not depend on a single optimal risk level, but can be realized through asymmetric combinations of different antecedent conditions. This possibility of multiple equifinal outcomes precisely corroborates the core proposition of flexibility in boundary management within edgework theory: individuals can obtain ideal boundary experiences through different risk-coping strategies and resource portfolios. At the same time, experience quality emerges as a core condition in all configurational paths, further consolidating the centrality of the affective hub in the CAB framework. In doing so, this study not only responds to the limitations of prior research that relied excessively on linear and net-effect analyses, but also advances risk perception and behavioral decision-making theories from a configurational perspective.

Third, at the methodological level, this study demonstrates the unique value of a mixed-methods approach combining structural equation modeling (SEM) and fuzzy-set qualitative comparative analysis (fsQCA) in adventure tourism safety research. SEM effectively tests the mediating and moderating mechanisms among variables, clarifying the net-effect relationships among risk perception, experience quality, and behavioral intention, as well as the moderating role of gender in these pathways. Meanwhile, fsQCA captures the conjunctural effects of multiple factors and equifinality characteristics from a holistic perspective. The complementarity of these two methods not only enhances the internal validity and external explanatory power of the research findings, but also provides a replicable analytical paradigm for future investigations into complex relationships among risk perception, affective experience, and behavioral decision-making.

### 5.6 Implications for governance

In the context of safety governance for mountain outdoor sports, adopting a “risk marginality” perspective is of significant value. This perspective emphasizes not only the elimination of risks but also the creation of an environment that balances challenge with safety through effective risk management.

First, strengthening integrated risk management serves as the foundation for effective safety governance. Destinations should prioritize the control of natural risks through environmentally adaptive design, reasonable activity route planning, and the implementation of meteorological monitoring systems to mitigate threats posed by weather and terrain. In addition, it is crucial to enhance team coordination and communication, equip sites with advanced rescue technologies and equipment, and ensure that gear meets safety standards to minimize the risks faced by tourists.

Second, attention should be paid to the interaction between tourists' risk perception and experience quality. Destinations should improve risk communication and education, enhancing tourists' skills and psychological coping abilities to strengthen their capacity to perceive and manage risks. Furthermore, improving service quality, infrastructure, and the overall enjoyment of activities can significantly enhance tourists' experience, thereby fostering stronger behavioral intentions.

Finally, safety management strategies should be tailored to account for gender differences. Destinations should design differentiated activities and safety measures that reflect the characteristics and needs of male and female tourists. For female tourists, who generally exhibit a higher tendency toward risk aversion, it is especially important to provide comprehensive safety assurances and emotional support.

### 5.7 Limitations and future research directions

This study has the following limitations. First, the sample is biased and geographically constrained. The data were collected exclusively from risk-preferring tourists at the Siguniang Mountain Scenic Area. A single destination and a sample with a specific risk orientation may weaken the generalizability of the conclusions to other regions, to low-risk-preference groups, and to different types of mountain adventure activities. Future research should expand sample heterogeneity, encompassing diverse tourist types and multiple geographic contexts, and conduct cross-sample comparisons. Second, the cross-sectional design makes it difficult to reveal dynamic causality. Risk perception and experience quality may change markedly during the pre-activity, activity, and post-activity stages, yet a single-time-point measurement cannot capture the time-lag effects and evolutionary processes. Subsequent studies can introduce longitudinal tracking or the experience sampling method to conduct multi-time-point observations across the entire process, thereby clarifying the temporal relationships and causal mechanisms among variables. Third, the completeness of the risk perception dimensions is insufficient. The existing dimensions may not cover important factors such as information risk and public health risk, and the seasonal fluctuations of mountain environments, which can alter the risk structure, were not considered. Future work should further enrich the dimensions and explore the relationships between risk configurations and behavioral intentions across different seasons. Fourth, the deeper mechanisms underlying gender differences require further investigation. The non-significant risk perception–experience quality–behavioral intention pathway in the female group suggests a distinct risk decision-making logic. Future research can employ qualitative methods to explore its socio-cultural causes and develop gender-sensitive management strategies.

## 6 Conclusion

In mountain outdoor sports, risk perception is not a threat to be minimized but a resource to be optimized. Rather than eliminating risk, destination managers should maintain it within an optimal range where it serves as a motivator rather than a deterrent. This shifts the paradigm from risk control to risk optimization.

First, from the perspective of risk marginality, risk perception has a significant positive effect on behavioral intention, and perceived experience quality acts as a partial mediator. Conceptually, this means that risk, when properly contextualized, transforms from a deterrent into an attractor—a finding that resolves the long-standing “risk paradox” in adventure tourism.

Second, gender significantly moderates these relationships, revealing that the risk-to-intention mechanism operates differently for male and female tourists. Conceptually, this underscores that risk perception is not gender-neutral; its motivational power is socially and psychologically gendered. Destination planners should therefore move beyond one-size-fits-all safety strategies and design gender-sensitive interventions.

Third, all four risk dimensions (individual, natural, social, equipment) positively affect behavioral intention, but individual and natural risks have the strongest effects. This hierarchy implies that risk governance should prioritize the “core edge” risks (personal capacity and environmental hazards) while not neglecting peripheral risks (social and equipment), as the latter still contribute positively through substitution effects revealed by fsQCA.

Finally, the fsQCA configurational analysis identifies two pathway types—(1) Individual–Natural Risk Intertwining and (2) Equipment–Experience Buffering—demonstrating equifinality. Conceptually, this means that no single risk dimension is indispensable; different combinations can compensate for each other. Destinations should design flexible risk management systems that allow for multiple pathways to high behavioral intention, rather than assuming a single optimal risk profile. The key takeaway is a shift from risk elimination to risk optimization. Destination managers should: (a) identify the optimal risk range for their specific context, (b) use experience quality as a buffer to transform perceived risk into positive affect, and (c) design flexible, gender-sensitive, and configurational safety strategies that leverage substitution effects among risk dimensions.

## Data Availability

The raw data supporting the conclusions of this article will be made available by the authors, without undue reservation.

## References

[B1] Barbieri C. Sotomayor S. (2013). Surf travel behavior and destination preferences: an application of the serious leisure inventory and measure. Tour. Manag. 35, 111–121. doi: 10.1016/j.tourman.2012.06.005

[B2] Berdychevsky L. Gibson H. J. (2015). Sex and risk in young women's tourist experiences: context, likelihood, and consequences. Tour. Manag. 51, 78–90. doi: 10.1016/j.tourman.2015.04.009

[B3] Brown L. de Coteau D. Lavrushkina N. (2020). Taking a walk: the female tourist experience. Tour. Stud. 20, 354–370. doi: 10.1177/1468797620930036

[B4] China Adventure Association (2025). The Alarm Bells Always Ring! The China Exploration and Research Society has Released The “2024 China Outdoor Adventure Accident Report”! Available online at: https://www.caa1993.org.cn/content/86/2925.shtml (Accessed January 24, 2025).

[B5] Crilly D. Zollo M. Hansen M. T. (2012). Faking it or muddling through? Understanding decoupling in response to stakeholder pressures. Acad. Manag. J. 55, 1429–1448. doi: 10.5465/amj.2010.0697

[B6] Doll W. J. Xia W. Torkzadeh G. (1994). A confirmatory factor analysis of the end-user computing satisfaction instrument. MIS Q 18, 453–461. doi: 10.2307/249524

[B7] Fave A. D. Bassi M. Massimini F. (2003). Quality of experience and risk perception in high-altitude rock climbing. J. Appl. Sport Psychol. 15, 82–98. doi: 10.1080/10413200305402

[B8] Fiss P. C. (2011). Building better causal theories: a fuzzy set approach to typologies in organization research. Acad. Manag. J. 54, 393–420. doi: 10.5465/amj.2011.60263120

[B9] Hongwei T. Linying X. Yimin H. Gongxing G. (2017). The effect of destination image on tourist behavior intention: an explanation based on the emotion appraisal theory. Tour. Trib. 32, 32–41.

[B10] Jincheng P. Hai L. (2020). Motivation, emotion andwillingness of adventure sports tourism under the marginal risk. J. Shanghai Univ. Sport 44, 34–42.

[B11] Jing L. Pearce P. L. Bihu W. Morrison A. M. (2015). The impact of smog on risk perception and satisfaction of international and domestic tourists in Beijing. Tour. Trib. 30, 48–59.

[B12] Kim J. Seo Y. (2019). An evolutionary perspective on risk taking in tourism. J. Travel Res. 58, 1235–1248. doi: 10.1177/0047287518807579

[B13] Laurendeau J. (2006). “He didn't go in doing a skydive”: sustaining the illusion of control in an edgework activity. Sociol. Perspect. 49, 583–605. doi: 10.1525/sop.2006.49.4.583

[B14] Lemke F. Clark M. Wilson H. (2011). Customer experience quality: an exploration in business and consumer contexts using repertory grid technique. J. Acad. Mark. Sci. 39, 846–869. doi: 10.1007/s11747-010-0219-0

[B15] Lepp A. Gibson H. (2003). Tourist roles, perceived risk and international tourism. Ann. Tour. Res. 30, 606–624. doi: 10.1016/S0160-7383(03)00024-0

[B16] Lyng S. (1990). Edgework: a social psychological analysis of voluntary risk taking. Am. J. Sociol. 95, 851–886. doi: 10.1086/229379

[B17] Meiyu W. Li C. Yong L. Feifeng C. Yunlang W. (2024). Because it's there: a complex deconstruction of the effects of the mountain explorer's drive and its intention to explore. Tour. Trib. 39, 103–120.

[B18] Moon H. Han H. (2019). Tourist experience quality and loyalty to an island destination: the moderating impact of destination image. J. Travel Tour. Mark. 36, 43–59. doi: 10.1080/10548408.2018.1494083

[B19] Muehling D. D. Sprott D. E. Sprott D. E. (2004). The power of reflection: an empirical examination of nostalgia advertising effects. J. Advert. 33, 25–35. doi: 10.1080/00913367.2004.10639165

[B20] Pappas I. O. Kourouthanassis P. E. Giannakos M. N. Chrissikopoulos V. (2016). Explaining online shopping behavior with fsQCA: the role of cognitive and affective perceptions. J. Bus. Res. 69, 794–803. doi: 10.1016/j.jbusres.2015.07.010

[B21] Park K. Reisinger Y. (2010). Differences in the perceived influence of natural disasters and travel risk on international travel. Tour. Geogr. 12, 1–24. doi: 10.1080/14616680903493621

[B22] Runze M. Yuan L. Zhen L. (2024). Influencing factors and configuration paths of camping tourists' behavioral intention—a qualitative comparative analysis based on fuzzy sets. Tour. Sci. 38, 88–108.

[B23] She S. Tian Y. Lu L. Eimontaite I. Xie T. Sun Y. (2019). An exploration of hiking risk perception: dimensions and antecedent factors. Int. J. Environ. Res. Public Health 16:1986. doi: 10.3390/ijerph16111986 31167460 PMC6603918

[B24] Tavitiyaman P. Qu H. (2013). Destination image and behavior intention of travelers to Thailand: the moderating effect of perceived risk. J. Travel Tour. Mark. 30, 169–185. doi: 10.1080/10548408.2013.774911

[B25] Wolff K. Larsen S. and O. Gaard T. (2019). How to define and measure risk perceptions. Ann. Tour. Res. 79:102759. doi: 10.1016/j.annals.2019.102759

[B26] Yanbo Y. Pingping H. (2019). A study on female tourism risk perception dimension. Consum. Econ. 35, 88–96.

[B27] Yang E. C. L. Khoo-Lattimore C. Arcodia C. (2017). A systematic literature review of risk and gender research in tourism. Tour. Manag. 58, 89–100. doi: 10.1016/j.tourman.2016.10.011

[B28] Yang Z. Xuegang F. (2023). Influence of sport tourism service quality on tourists' experience quality and behavior intention. J. Shanghai Univ. Sport 47, 57–71.

[B29] Yangle C. Wenxin C. (2023). Impact of risk perception on surfers' intention to revisit is mediated by emotion and experience quality. Trop. Geogr. 43, 2024–2034.

[B30] Yong L. Dan L. Zhaofeng G. Jiayang Z. Erwei D. WeiZhong Z. (2025). Risk and reward: a study on the influencing mechanism of decision-making behavior of Mountaineering in Siguniang mountain region. J. Outdoor Recreat. Tour. 51:100895. doi: 10.1016/j.jort.2025.100895

[B31] Yüksel A. Yüksel F. (2007). Shopping risk perceptions: effects on tourists' emotions, satisfaction and expressed loyalty intentions. Tour. Manag. 28, 703–713. doi: 10.1016/j.tourman.2006.04.025

[B32] Zhaofang P. Hongyou L. Wei G. Xiaoyang C. Cencen T. Yuhui J. . (2018). A study of the building of a mountain outdoor sports risk evaluation index system and an early warning system in China. J. Phys. Educ. 25, 68–73.

